# Subthalamic Nucleus Activity during Cognitive Load and Gait Dysfunction in Parkinson's Disease

**DOI:** 10.1002/mds.29455

**Published:** 2023-05-25

**Authors:** Matthew J. Georgiades, James M. Shine, Moran Gilat, Jacqueline McMaster, Brian Owler, Neil Mahant, Simon J.G. Lewis

**Affiliations:** ^1^ ForeFront Parkinson's Disease Research Clinic, Brain and Mind Centre University of Sydney Sydney New South Wales Australia; ^2^ Sydney Medical School The University of Sydney Sydney New South Wales Australia; ^3^ KU Leuven, Department of Rehabilitation Sciences Neurorehabilitation Research Group (eNRGy) Belgium; ^4^ Westmead Private Hospital Sydney New South Wales Australia

**Keywords:** freezing of gait, Parkinson's disease, subthalamic nucleus, deep brain stimulation, virtual reality

## Abstract

**Background:**

Gait freezing is a common, disabling symptom of Parkinson's disease characterized by sudden motor arrest during walking. Adaptive deep brain stimulation devices that detect freezing and deliver real‐time, symptom‐specific stimulation are a potential treatment strategy. Real‐time alterations in subthalamic nucleus firing patterns have been demonstrated with lower limb freezing, however, whether similar abnormal signatures occur with freezing provoked by cognitive load, is unknown.

**Methods:**

We obtained subthalamic nucleus microelectrode recordings from eight Parkinson's disease patients performing a validated virtual reality gait task, requiring responses to on‐screen cognitive cues while maintaining motor output.

**Results:**

Signal analysis during 15 trials containing freezing or significant motor output slowing precipitated by dual‐tasking demonstrated reduced θ frequency (3–8 Hz) firing compared to 18 unaffected trials.

**Conclusions:**

These preliminary results reveal a potential neurobiological basis for the interplay between cognitive factors and gait disturbances including freezing in Parkinson's disease, informing development of adaptive deep brain stimulation protocols. © 2023 The Authors. *Movement Disorders* published by Wiley Periodicals LLC on behalf of International Parkinson and Movement Disorder Society.

Freezing of gait (FOG) in Parkinson's disease (PD) is a common and disabling symptom characterized by sudden, paroxysmal motor arrest during walking that often leads to falls and poor quality of life.^1^ There are various observed triggers for freezing including dual‐tasking, where additional cognitive demands are imposed during walking.^2^, ^3^ Patients with PD and FOG have impaired cognitive and inhibitory control, particularly during cognitive load.^4^, ^5^ It has been suggested that pathophysiology in fronto‐subthalamic circuits within the cognitive control network^6^ may contribute to FOG.^7^ Functional neuroimaging has demonstrated decoupling within this network in PD patients with FOG during high cognitive load.^8^ Furthermore, microelectrode recordings obtained during deep brain stimulation (DBS) surgery identified altered subthalamic nucleus (STN) firing coincident with lower limb freezing during virtual reality (VR) gait task performance.^9^ This suggests FOG pathogenesis involves an interplay between cognitive and motor factors within cortico‐basal ganglia circuits, potentially converging in the STN. To date, STN activity patterns between effective and dysfunctional motor‐cognitive dual‐tasking in PD have not been examined, limiting the development of adaptive DBS systems toward targeting freezing in a variety of contexts.

We used a validated intraoperative VR gait paradigm^14^, ^15^ to explore STN activity in PD patients during periods of motor output with concurrent cognitive load. We sought to identify alterations in STN firing during cognitive cue processing that occurred with disruption of ongoing motor output (“gait” dysfunction) compared to periods where motor output and associated VR gait progression were unaffected by cognitive dual‐tasking.

## Methods

### Participants and Clinical Assessments

Eight patients were recruited through the ForeFront Parkinson's Disease Research Clinic, University of Sydney and Westmead Private Hospital with Human Research Ethics Committee approval. Written informed consent was obtained according to the Declaration of Helsinki. Patients were not selected for the presence of FOG, although patients who experienced freezing during the VR task all had FOG preoperatively.

### Intraoperative VR Gait Paradigm and Cognitive Dual‐Task

**FIG 1:**
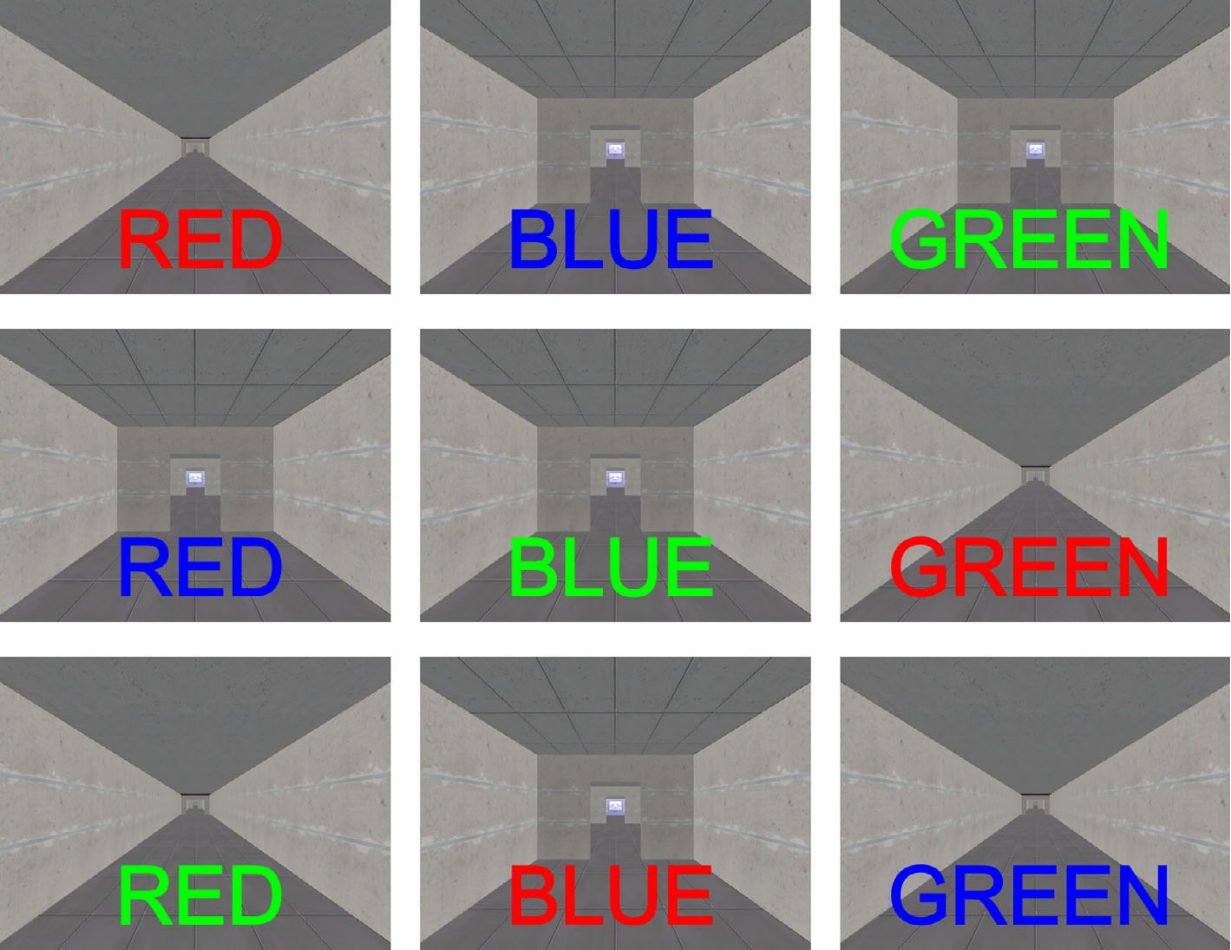
Display of cognitive cues presented during virtual reality (VR) gait task. Upper row displays congruent color‐word pairs for which patients were instructed to respond by continuing with their VR walking. The lower two rows contain incongruent color‐word pairs for which the patients were instructed to stop. After stopping, patients were instructed to recommence walking when an explicit “WALK” cue appeared on the screen within a variable 3–5 second period to avoid anticipation. [Color figure can be viewed at wileyonlinelibrary.com]

The intraoperative setup and VR gait task^4^, ^14^, ^15^, ^16^ were as described in previous work.^9^ Participants were *off* dopaminergic medication for ≥12 hours. Patients used foot pedals to navigate a VR environment displayed in first‐person view on a 40‐inch screen while lying supine on the operating table (Supplementary Fig. S2). The testing paradigm included cues requiring cognitive input from participants that appeared in the bottom third of the screen (Fig. 1) and consisted of pseudorandomized color‐word pairs that were either congruent (eg, “BLUE” written in blue text) or incongruent (eg, “RED” written in green text).^14^, ^15^ Subjects were instructed to keep walking for congruent color‐word cues and to stop for incongruent cues. Only appropriate responses to congruent (keep walking) cues were included in the analysis because we were specifically only interested in STN activity with cognitive load and ongoing motor output, and any incongruent (stop) segments would be confounded by STN activity associated with purposeful stopping. Overall, the task took between 2 and 3 minutes to complete with a minimum of 100 steps and 4 or 5 cognitive cues faced.

### Measures of VR Gait Performance with Cognitive Load

The timepoints of each foot pedal depression were recorded, along with foot pedal amplitude as a percentage of maximum range. The modal inter‐footstep latency (mFSL) was taken as a measure of typical latency between sequential VR footsteps for each patient computed from blocks free of “contaminants” (eg, cognitive cues, freezing, and start/stop cues).^9^, ^14^, ^15^ Foot pedal velocity (FPV) was calculated by taking the first derivative of foot pedal amplitude over time (dA/dt). Further details are provided in the Supplementary Data **S1**.

Maintaining validated definitions of VR motor arrests, a “freeze” was defined as any footstep latency >2× a participant's mFSL.^9^, ^14^, ^15^ Significant VR gait slowing (vs. normal progression) following cognitive cue presentation was pre‐defined as >50% reduction in FPV compared to pre‐cue footsteps. Episodes of cognitive cue‐associated freezing and VR gait slowing were pooled and compared against segments where VR gait metrics were unaffected by concurrent cognitive processing.

### Neurosurgical STN Recordings

Please refer to the Supplementary Data **S1**.

### Signal Processing

A time series of smoothed standardized STN multi‐unit activity (MUA) firing rate was computed according to an established protocol to facilitate permutation statistics.^9^ Further details are included in the Supplementary Data **S1**.

### Time‐Frequency Analysis

Data segments were aligned to cognitive cue presentation. Following previous approaches,^9^, ^19^, ^20^, ^21^, ^22^ the θ (3–8 Hz), α (9–12 Hz), β (13–30 Hz), and γ (40–150 Hz) modulation of the MUA signal was computed using a 2nd‐order Butterworth band‐pass filter. The envelope of θ, α, β, and γ modulation was calculated by passing these respective time series through a low‐pass (<4 Hz) filter.

### Statistical Analysis

Please refer to the Supplementary Data **S1**.

## Results

Please see the Supplementary Data **S1** for results of VR gait task performance parameters. There were 33 trials of congruent cognitive cue responses during VR gait. In 18 of these trials, motor output was not affected. The remaining 15 trials were pooled and classed as cognitive cue‐associated VR freezing (n = 8) or significant VR gait slowing where there was >50% FPV reduction (n = 7).

**FIG 2:**
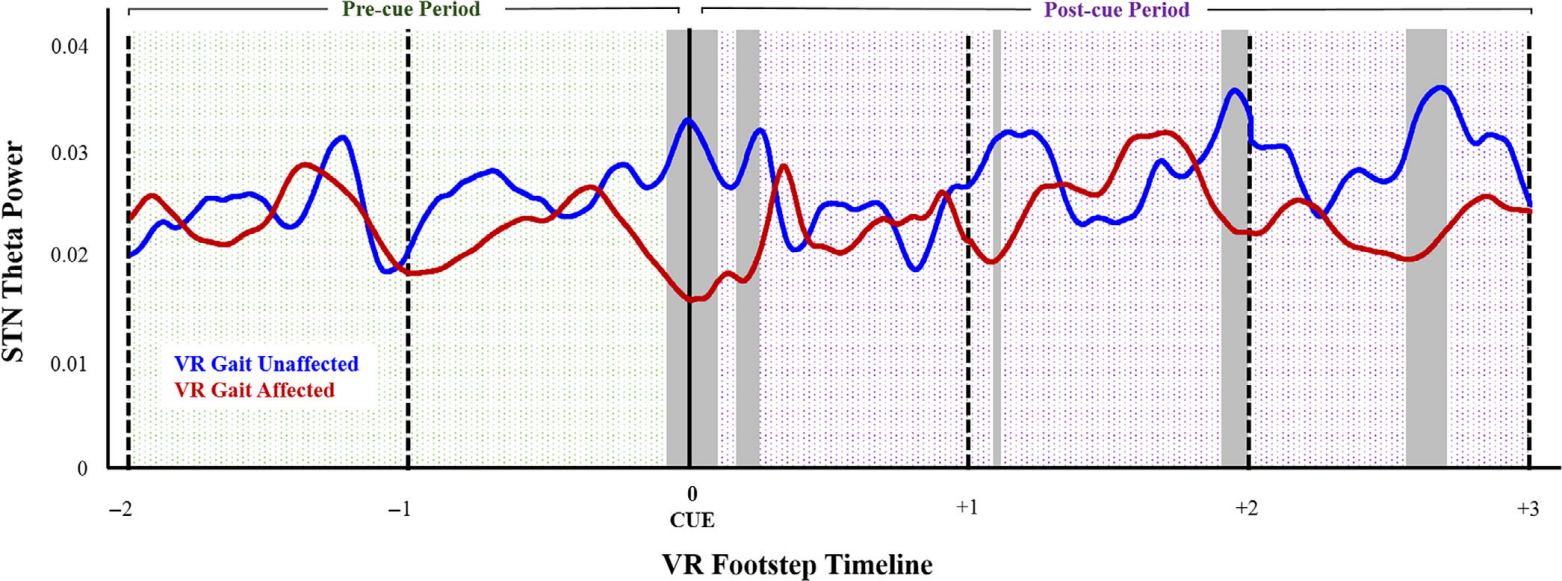
Subthalamic nucleus (STN) θ activity changes observed with cognitive dual‐tasking. θ frequency (3–8 Hz) modulation (power) of the group‐level mean MUA signal (y‐axis) is plotted over time (x‐axis, virtual reality [VR] footsteps). There was an observed reduction in STN θ (3–8 Hz) activity at the moment of cognitive cue presentation and shortly after for trials that evolved into freezing of gait or significant VR gait disturbance (red = VR gait affected) compared to those trials where VR gait progression was unaffected by cognitive cue processing (blue). There were also relative reductions in θ activity in close proximity to ensuing footsteps in these affected trials. The data has been aligned to cognitive cue presentation (footstep 0) and spans from 2 footsteps where subjects are walking before cue presentation (pre‐cue period = shaded green) to 3 footsteps after cue presentation (post‐cue period = shaded purple). Data has been scaled with linear interpolation to standardize each inter‐footstep latency to a segment of 1000 data points so that footsteps remain aligned. Shaded gray bars designate periods where differences in STN θ activity between trials was statistically significant. Results obtained using nonparametric statistical permutation testing with a significance level set to *P* = 0.05 with a correction for multiple comparisons. [Color figure can be viewed at wileyonlinelibrary.com]

Statistical permutation testing revealed a sustained and significant reduction in θ frequency STN firing centered on cognitive cue presentation in trials that evolved into lower limb freezing or significant VR motor output slowing compared to trials where VR gait progression was unaffected by cognitive load (Fig. 2). No significant differences were observed for other frequencies (Supplementary Fig. S3). We observed transient increases in STN MUA firing rate during the interval between cue presentation and the first post‐cue footstep in trials that contained freezing or VR gait slowing.

## Discussion

We report real‐time changes in STN MUA that occurred when progression of ongoing VR motor output was disturbed by concurrent cognitive processing. Specifically, we observed reduced θ activity coincident with cue presentation for trials where ongoing VR gait was disturbed by concurrent cognitive processing (either VR freezing or >50% FPV reduction), compared to trials that were unaffected by dual‐tasking (Fig. 2).

Recent work established a link between rhythmic cortical and subcortical θ activity and the dynamic allocation of attentional resources toward relevant environmental information.^25^ Reduced θ activity at cognitive cue presentation may be inadequate to reallocate attentional resources for effective gait maintenance with concurrent cognitive load, resulting in increased stepping variability, reduced velocity, and ultimately FOG.^7^ This is supported by functional neuroimaging studies demonstrating poor recruitment within the cognitive control network during periods of high cognitive load with VR gait in freezers.^26^ Another study observed increases in 7 to 12 Hz pedunculopontine activity (overlapping our θ range) that correlated with gait and cognitive performance and is thought to support the role of these frequencies in maintaining attention.^27^, ^28^

The emergence of STN DBS therapy in PD has enabled interrogation of neuronal oscillatory dynamics within neurophysiology and disease.^29^, ^30^ STN β and θ activity are understood to be required for suppression of pre‐potent responses during decision making and cognitive control.^6^, ^30^ STN θ power increases with cognitive processing, and when intentionally disrupted by STN stimulation, results in an increased error rate during high conflict cognitive trials.^31^ Impaired θ frequency synchronization in the STN of patients with PD also correlates with longer reaction times and cognitive errors.^32^ Taken together, our observations are consistent with the hypothesis that adequate STN θ synchrony is required for timely and accurate responses to cognitive tasks and the reduction of θ activity with cue presentation observed in trials where VR gait progression was subsequently disturbed, may relate to impaired cognitive dual‐tasking.

We recently reported significant increases in STN θ activity immediately before freeze onset in subjects performing the VR task, and to a lesser degree, with volitional stopping.^9^ Further research is required to explore whether the presently reported reduction in θ frequency activity relates specifically to altered cognitive processing or to freezing itself. The absence of previously demonstrated changes in STN β activity associated with freezing^9^ may potentially relate to inter‐trial variability in cognitive processing time, footstep latency, or the small dataset. We previously hypothesized that freezing should occur secondary to increased multi‐unit activity firing rate in the STN, mediated via conflict in cortical networks triggering a hyperdirect pathway.^8^, ^9^ This STN activity drives the globus pallidus internus to inhibit the pedunculopontine nucleus, impairing the coordination of central pattern generators in the spinal cord, culminating in dysfunctional firing of paired agonist–antagonist lower limb muscles.^8^ Conflict arising from dysfunctional cognitive processing may be one of many triggers for this cascade of abnormal firing, and reduced STN θ activity may represent a targetable substrate in DBS applications.

Closed loop adaptive DBS might represent an effective treatment strategy with dynamic stimulation parameters modulated in response to real‐time monitoring to abort a freeze.^33^, ^34^, ^35^, ^36^ This would require detection of signals that reliably precede FOG episodes, including those related to cognitive dual‐tasking. A remaining challenge for future work is to confirm our preliminary findings in a larger cohort and discern robust features that distinguish pathological freezing and gait disturbances with cognitive dual‐tasking from volitional stopping.

The current study is potentially limited by the surgically mandated supine positioning of the patients, which minimizes any contribution related to anticipatory postural adjustments along with the anxiety of potential falls.^37^ Another challenge lies in translating these findings to the development of adaptive DBS systems, which use implanted macroelectrodes to monitor local field potential (LFP) signals and are poorly suited to the acquisition of MUA.^34^ The relationship between LFP and MUA signals is not fully understood, but it may be possible to characterize the coherence between them, which could assist in overcoming this challenge along with novel DBS devices capable of recording additional signal types.^18^, ^34^, ^38^ Future work should obtain recordings from implanted DBS systems postoperatively in ambulant patients undergoing concurrent cognitive dual‐tasking, which should also guide reconciliation with previous work in LFPs.^30^, ^31^, ^32^

In conclusion, we have observed changes in STN activity that appear to be related to the phenomenon of dual‐task freezing in PD. By demonstrating alterations in STN firing patterns linked to gait dysfunction with cognitive dual‐tasking, we provide preliminary evidence for an accessible locus within the wider neurobiological substrate underpinning the phenomenon of cognitive dual‐task provoked freezing and gait dysfunction in PD. Given the prominent and well‐evidenced interplay between cognitive factors and gait disturbances in PD, this has implications for future therapies such as adaptive closed loop DBS systems that may be able to target this and improve quality of life for many patients.

## Author Roles

(1) Research Project: A. Conception and Design; B. Acquisition of Data; C. Analysis and Interpretation of Data; D. Neurosurgical Operation; E. Intraoperative Neurological Assessment; F. Principal Investigator (2) Manuscript: A. Writing of the First Draft; B. Review and Critique.

M.J.G.: 1A, 1B, 1C, 2A.

J.M.S.: 1A, 1B, 1C, 2B.

M.G.: 1B, 2B.

J.M.: 1D, 2B.

B.O.: 1D, 2B.

N.M.: 1A, 1B, 1E, 2B.

S.J.G.L: 1A, 1F, 2B.

## Financial Disclosures

ForeFront is a collaborative research group at the Brain and Mind Centre, University of Sydney. We thank the support from National Health and Medical Research Council (NHMRC) program (1132524), Dementia Research Team (1095127) and NeuroSleep Centre for Research Excellence (1060992) grants. M.J.G. was supported by the RA Money Postgraduate Research Scholarship in Neuroscience, University of Sydney. J.M.S. is supported by a NHMRC Fellowship (1193857). M.G. is funded by the Marie Sklodowska‐Curie Actions grant agreement (838576). S.J.G.L. is supported by a NHMRC Leadership Fellowship (1195830). The funding sources had no role in study design, data collection, analysis, interpretation, or manuscript preparation.

## Supporting information


**Data S1.** Supporting information.

## Data Availability

The data that support the findings of this study and custom code used for analyses are available from the corresponding author upon reasonable request. The data are not publicly available as they contain information that could compromise research participant privacy.
