# Supplementary material for: Subthalamic Nucleus Activity during Cognitive Load and Gait Dysfunction in Parkinson's Disease
Source: Mov Disord. 2023 May 25;38(8):1549–54. doi: 10.1002/mds.29455 (PMC10946988; doi:10.1002/mds.29455)
Supplement: Supplementary file 1 — Data S1. Supporting information. [file MDS-38-1549-s001.docx]

**Supplement to Methods**

Participants and clinical assessments

Participants underwent preoperative neurological assessment and diagnosis of idiopathic PD according to United Kingdom Parkinson’s Disease Society Brain Bank criteria.^10^ Assessments are summarized in Supplementary Table S1.^11-13^

Intraoperative virtual reality gait paradigm and cognitive dual task

Forward progression through the VR environment and corresponding on-screen movement required alternating left-right foot pedal depressions beyond a 30 degree threshold from the resting position. Software configuration prevented progression for consecutive unilateral depressions (e.g. left-left), subthreshold depressions and incomplete pedal release, however all inputs were recorded for analysis.

We identified 37 data segments consisting of three VR footsteps pre- and post- cognitive cue presentation for analysis. Only segments for congruent (i.e., “keep walking”) cues were extracted in order to explore STN signal changes with variations in ongoing VR gait performance subsequent to cognitive cue processing. Four trials were excluded due to the subjects’ feet sliding off the footpedals near cognitive cue presentation invalidating the data, leaving 33 trials.

The coefficient of variation (calculated as the quotient of the sample standard deviation and sample mean COV = StDev/Mean) was also taken as a measure of gait progression and rhythmicity and compared for the three steps pre- and post-cue.

Neurosurgical STN recordings

As per standard of care, patients were awake during surgery. Target coordinates and trajectory planning were determined by stereotactic co-registration of preoperative T2-weighted MRI images with Brain Lab navigation planning software. Intraoperatively, the desired STN recording site was identified according to stereotactic coordinates along the implantation trajectory and confirmed by a neurophysiologist (NM) as a microTargeting™ Electrode (FHC Inc., Bowdoin, ME, USA) was advanced from 10mm above the surgical target according to established protocols.^9^ The microelectrode obtained recordings of extracellular pooled cell body action potentials from STN neurons (multi-unit activity, MUA) and transmitted these via a Medtronic LeadPoint® amplifier (1,000 MΩ input impedance). The amplified signal was passed through an adaptive 50 Hz line filter and bandpass filtered (200-5,000 Hz) and digitized at 25 kHz. The VR gait task was commenced (and recordings simultaneously collected) intraoperatively in the uppermost 1-2mm of STN contralateral to the individual’s most affected side. As previously described^9^, the extracellular recording of pooled cell body action potentials in the immediate vicinity of the microelectrode is akin to placing a microphone in a room hosting a cocktail party. This enables observation of the ongoing surrounding ‘conversations’ of nearby neurons (i.e. an ensemble of MUA), without focusing precisely on who said what (i.e. the firing of individual subthalamic nucleus neurons). Single cell microelectrode recording was not attempted due to the difficulty of maintaining prolonged stable recordings in an awake, actively moving human.

Signal processing

Analyses were performed off-line using MATLAB R2019a (MathWorks, MA, USA). The interrogation of freezing neurobiology via analysis of LFP signals has been popularized in a number of studies, likely due to its ease of acquisition from implanted macroelectrodes compared to the technical challenges of obtaining intraoperative microelectrode recordings required for capture of MUA signals. However its interpretation in the causative pathophysiology of freezing is limited by the fact it is recorded from a relatively large volume of neuronal material and its signal is therefore dominated by the pooled membrane currents of synaptic inputs rather than cell body output activity.^17^ Examination of pooled neuronal cell body action potentials represented by the MUA signal captured by intraoperative microelectrode recordings therefore affords novel and potentially more interpretable insights along with a superior means for directly testing the hypothesis that pathological subthalamic nucleus output activity mediates the freezing behaviour associated with cognitive load in Parkinson’s disease.^18^

We applied a 150 Hz high-pass second order Butterworth filter passed forwards and backwards^9^. A threshold value of two standard deviations above the mean signal was selected to obtain a timeseries of pooled, above-baseline MUA firing. An instantaneous measure of STN MUA firing rate was computed by taking the inverse of the latency between supra-threshold data points in the MUA signal and multiplying by the sample rate (25 kHz). This signal was smoothed with a 1 ms sliding window before individual subject smoothed signals were standardized to the respective signal range (x’ = [x – min(x)]/[max(x) – min(x)]), to permit grouped statistics. The individual standardized and smoothed MUA firing rate timeseries were then aligned with 33 cognitive cue presentation events and segments were extracted corresponding to cue ± three VR footsteps.

To account for variable segment length resulting from inter-subject and inter-event variability in both VR footstep latency and duration of VR cognitive cue responses, data segments were down-sampled and linear interpolation standardized each inter-footstep latency to a segment of 1,000 data points. This enabled inter-trial alignment of data and group-level permutation statistics.

Statistical analysis

Paired t-tests were used to compare VR gait parameters between the three pre- and post- cognitive cue footsteps. STN MUA firing rate, along with theta, alpha, beta and gamma activity were compared between pre- and post- cognitive cue periods using nonparametric permutation testing with 5,000 permutations and a significance level *p* = 0.05, testing the proportion of null permutations in which a randomized dataset had a greater between-trial mean than the experimental data.^23^ A multiple comparisons correction popularized in EEG data analysis was applied, which only permitted significant values if neighboring temporal regions were also statistically significantly different.^24^ Summary data are presented as mean ± standard error of the mean (SEM).

**Supplement to Results**

Virtual Reality gait task performance

There were 33 trials of congruent cognitive cue responses during VR gait. In 18 of these trials, there was no significant difference in FSL variability between pre-cue (Mean COV_FSL_ = 0.10 ± 0.06) and post-cue (Mean COV_FSL_ = 0.11 ± 0.080) footsteps; t(17) = -1.030, *p* = 0.16, nor was there a significant difference in FPV variability between pre-cue (Mean COV_FPV_ = 0.16 ± 0.005) and post-cue (Mean COV_FPV_ = 0.18 ± 0.007) footsteps; t(17) = -1.10, *p* = 0.14. Hence in these 18 trials, VR motor output was not significantly affected by concurrent cognitive processing. The remaining 15 cognitive cue trials were classed as cognitive cue-associated VR freezing (n = 8) or significant VR gait slowing where there was >50% FPV reduction (n = 7). In these trials there was a significant increase in FSL and FPV variability from the pre-cue (Mean COV_FSL_ = 0.16 ± 0.02; Mean COV_FPV_ = 0.22 ± 0.01) to the post-cue (Mean COV_FSL_ = 0.50 ± 0.06; Mean COV_FPV_ = 0.30 ± 0.01) condition; t(14) = -6.26, *p* < 0.001; t(14) = -2.09, *p* = 0.03 respectively. In the 15 trials containing cognitive dual task associated-VR gait disturbances, variability was increased for the 3 pre-cue footsteps (COV_FSL_ = 0.16 ± 0.02 and COV_FPV_ = 0.22 ± 0.01) compared with the 3 pre-cue footsteps in the 18 trials with unaffected VR gait (COV_FSL_ = 0.10 ± 0.01; t(20) = -1.82, *p* = 0.04 and COV_FPV_ = 0.16 ± 0.01; t(23) = -1.78, *p* = 0.04).

| Patient | Age (years) | Disease duration (years) | UPDRS-III OFF | UPDRS-R | LED (mg/day) | FOGQ  Total/Q3 | mFSL  (msec) | # Trials VR unaffected post cog cue | # Trials VR FOG/slowing post cog cue |
| --- | --- | --- | --- | --- | --- | --- | --- | --- | --- |
| 1 (M) | 71 | 5 | 47 | 27 | 1200 | 1 / 0 | 500 | 1 | 2/1 |
| 2 (F) | 72 | 11 | 47 | 37 | 1120 | 4 / 0 | 1300 | 5 | 0/0 |
| 3 (F) | 62 | 7 | 35 | 36 | 1100 | 6 / 1 | 700 | 3 | 0/2 |
| 4 (F) | 64 | 12 | 51 | 51 | 800 | 12 / 2 | 1200 | 3 | 0/0 |
| 5 (M) | 67 | 13 | 43 | 45 | 1200 | 13 / 3 | 800 | 1 | 2/0 |
| 6 (M) | 67 | 6 | 58 | 39 | 800 | 3 / 0 | 700 | 1 | 2/2 |
| 7 (M) | 57 | 14 | 67 | 62 | 900 | 15 / 3 | 900 | 1 | 1/1 |
| 8 (M) | 72 | 4 | 68 | 36 | 900 | 3 / 0 | 500 | 3 | 1/1 |

**Supplementary Table S1. Clinical details of study participants** Participants included in this study are the same as those from Georgiades MJ *et al.* 2019 [9]. UPDRS-III = Movement Disorders Society Unified Parkinson’s Disease Rating Scale motor subscore (part III); UPDRS-R = Movement Disorders Society Unified Parkinson’s Disease Rating Scale remaining subscore (parts I,II,IV); LED = Levodopa Equivalent Dose (mg/day); FOG-Q = Freezing of Gait Questionnaire (total score/24); FOG-Q3 = Freezing of Gait Questionnaire item 3 (score/4); mFSL = VR gait task modal footstep latency (msec); The final two columns display the numbers of cognitive cue trials where VR gait was unaffected and then the number of trials where either FOG or significant VR gait slowing occurred in the three footsteps post cognitive cue presentation. All subjects that experienced freezing episodes during VR gait task performance also had documented freezing as observed by a clinician during pre-operative clinical assessments.


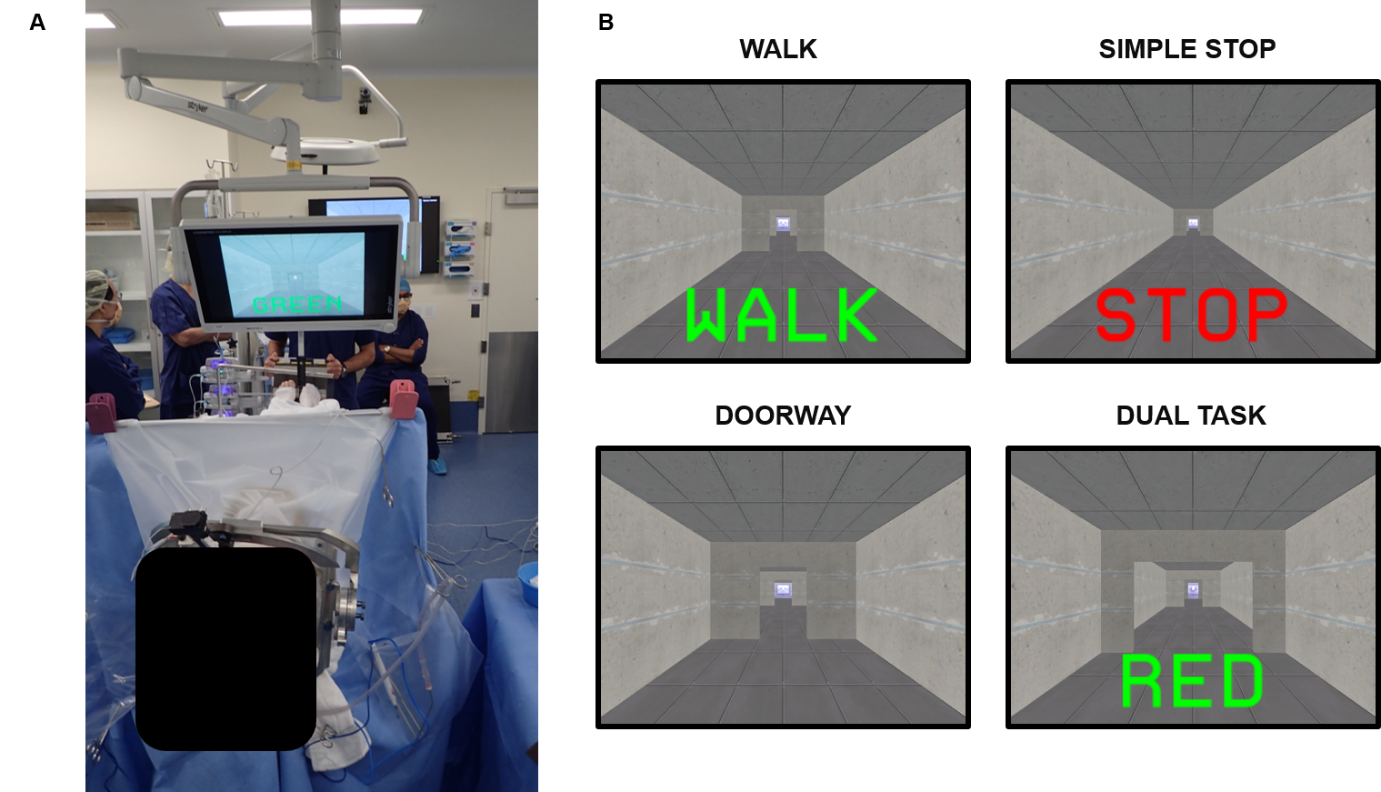


**Supplementary Figure S2. Intraoperative experimental setup with a congruent cognitive cue on screen.**


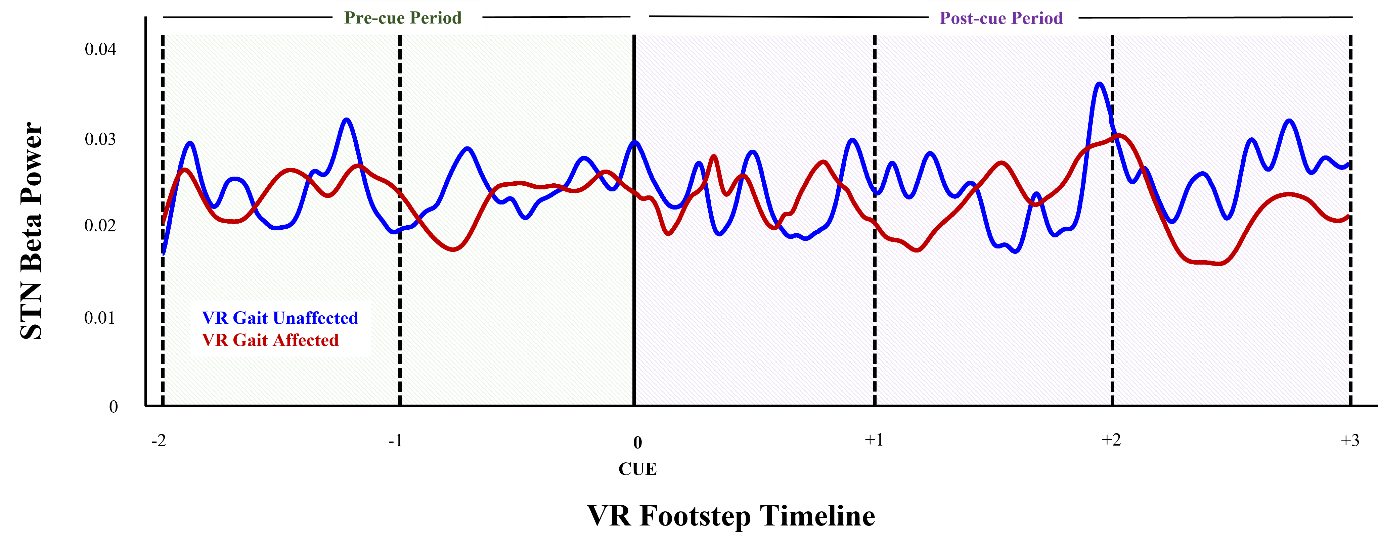
**Supplementary Figure S3.** **STN beta activity changes observed with cognitive dual tasking.** Beta frequency (13-30 Hz) modulation (power) of the group-level mean MUA signal (y-axis) is plotted over time (x-axis, VR footsteps). There was no observed statistically significant difference in STN beta activity between trials where VR gait progression was (red = VR gait affected) and was not (blue = VR gait unaffected) affected by cognitive cue processing. There was also no observed statistically significant difference in STN alpha nor gamma frequency activity (not pictured). The data has been aligned to cognitive cue presentation (footstep 0) and spans from 2 footsteps where subjects are walking prior to cue presentation (Pre-cue Period = shaded green) to 3 footsteps after cue presentation (Post-cue Period = shaded purple). Data has been scaled with linear interpolation in order to standardize each inter-footstep latency to a segment of 1,000 data points so that footsteps remain aligned. Results obtained using nonparametric statistical permutation testing with a significance level set to *p* = 0.05 with a correction for multiple comparisons.
